# Retrospective Cohort Analysis of Treatment Patterns, Survival, and Cost‐Effectiveness in Relapsed/Refractory Diffuse Large B‐Cell Lymphoma in Lower Austria (2018–2022)

**DOI:** 10.1002/cam4.71667

**Published:** 2026-02-24

**Authors:** Josef Singer, Sandra Gottsauner‐Wolf, Doris A. Behrens

**Affiliations:** ^1^ Karl Landsteiner University of Health Sciences Krems Austria; ^2^ Department of Internal Medicine II University Hospital Krems, Karl Landsteiner University of Health Sciences Krems Austria; ^3^ Strategy and Quality Medicine – Medical Strategy and Development Landesgesundheitsagentur Niederösterreich St. Pölten Austria; ^4^ Department for Economy and Health University for Continuing Education Krems Krems Austria; ^5^ Employee Wellbeing Service Aneurin Bevan University Health Board Cwmbrân UK; ^6^ School of Mathematics Cardiff University Cardiff UK

**Keywords:** bispecific antibodies, CAR‐T cell therapy, cost‐effectiveness, incremental cost‐effectiveness ratio, real‐world evidence, relapsed/refractory diffuse large B‐cell lymphoma

## Abstract

**Background:**

R/R DLBCL is an aggressive malignancy with limited treatment options. Novel immunotherapies have improved outcomes in selected clinical‐trial populations, but their effectiveness in real‐world settings remains unclear. Their substantial costs also pose challenges for healthcare systems.

**Methods:**

This exploratory study evaluated the efficacy and costs of available therapies in a real‐world population. Two retrospective analysis approaches were used: First, we compared calendar‐based cohorts: A conventional‐treatment period (1 January 2018–31 December 2019), which predates EMA approval of Polatuzumab‐vedotin, and a modern‐treatment period (1 January 2020–31 December 2022). Second, because treatments overlapped across periods, we additionally grouped patients by therapy exposure and compared those receiving any modern therapy at any stage with those treated exclusively with conventional regimens.

**Results:**

Comparing the calendar‐based periods provided limited insights, as therapies unavailable in the earlier period could still be used in subsequent treatment lines. In contrast, grouping patients by exposure to modern therapy at any stage showed a clear survival benefit: Median OS was 20 vs. 5 months (95% CI: 1.8–38.2 vs. 3.0–7.0; *p* = 0.022). This advantage was associated with significantly higher mean costs (€178,513.08 vs. €15,185.08; *p* < 0.001), resulting in an incremental cost of €10,889 per additional month of survival.

**Conclusion:**

Modern therapies for r/r DLBCL have significantly improved survival rates, but their high costs necessitate careful cost‐effectiveness assessments to ensure they are integrated optimally into clinical practice. Additionally, our findings indicate that calendar‐based comparisons can be misleading, since novel treatments do not simply “start” and “stop” at approval dates—a crucial methodological insight that is highly generalizable to real‐world evidence studies. Grouping patients by actual therapy exposure offers a more accurate evaluation of clinical benefits and economic impact when new treatments become routine. Achieving equitable access to treatment while maintaining healthcare sustainability will require coordinated efforts among clinicians, researchers, and policymakers.

**Abbreviations**
n.a: not applicable
R^2^
: rituximab, lenalidomide
R‐CHOP: rituximab, cyclophosphamide, doxorubicin, vincristine, prednisone
R‐COMP: rituximab, cyclophosphamide, liposomal doxorubicin, vincristine, prednisone
R‐DHAP: rituximab, dexamethasone, high‐dose cytarabine, cisplatin
R‐EPOCH: rituximab, etoposide, cyclophosphamide, doxorubicin, vincristine, prednisone
R‐GemOx: rituximab, gemcitabine, oxaliplatin
R‐ICE: rituximab, ifosfamide, carboplatin, etoposide

## 1 Introduction

Relapsed or refractory diffuse large B‐cell lymphoma (r/r DLBCL) is an aggressive cancer with limited treatment options [1]. Although first‐line chemoimmunotherapy with R‐CHOP induces remission in about 60% of patients, outcomes remain poor for those with relapsed or refractory disease [2]. Until recently, high‐dose salvage chemotherapy followed by autologous stem cell transplantation (ASCT) was the only potentially curative second‐line option—a strategy often unsuitable due to patient age, frailty, or resistance to chemotherapy [3].

In recent years, novel immunotherapies have transformed the treatment landscape of r/r DLBCL. A pivotal milestone was the EU approval of the CD79b‐targeting antibody–drug conjugate polatuzumab vedotin in 2020, which effectively marked the turning point in the treatment of r/r DLBCL and introduced a new generation of more targeted and less toxic therapies into routine care [4]. This was followed by Fc‐enhanced monoclonal antibodies such as tafasitamab [5], chimeric antigen receptor (CAR) T‐cell therapies [6, 7, 8, 9], and bispecific antibodies—also known as T‐cell engagers—which simultaneously bind tumor and T cells to redirect cytotoxic activity (e.g., epcoritamab and glofitamab) [10, 11]. These agents have demonstrated high response rates and durable outcomes across multiple clinical trials, including in patients ineligible for ASCT or with multiple prior relapses (see Supplementary Table S1 for a summary of clinical trial data).

Compared to conventional chemotherapy, these immunotherapies offer substantial clinical benefit—yet their integration into routine care presents challenges.

First, clinical trials are conducted in controlled settings with highly selected study populations, often excluding older or comorbid individuals. This limits the generalizability of trial findings to everyday clinical practice.

Second, financial toxicity is increasingly recognized as a key challenge in oncology [12]. This concept describes the financial strain that cancer treatment can place on patients and their families, including out‐of‐pocket costs, travel expenses, lost income, and employment disruptions. These pressures heighten the individual burden of illness and exemplify broader concerns about health equity [13].

Third, the excessive costs of acquiring these therapies raise concerns about affordability and sustainability, especially in publicly funded health systems. Outside of sponsored clinical trials, this risks patients in need of innovative treatments being unable to access them.

Cost‐effectiveness analyses (CEAs) provide a structured framework for evaluating the balance between therapeutic benefit and cost [14]. A key metric in this context is the incremental cost‐effectiveness ratio (ICER), which measures the additional cost per additional unit of health benefit when comparing two interventions [15]. The most widely accepted measure of health benefit in this context is the quality‐adjusted life year (QALY), which combines both survival and patient‐reported quality of life into a single value ranging from 0 (equivalent to death) to 1 (perfect health) [16].

Importantly, cost‐effectiveness is not merely a technical matter. It entails fundamental ethical questions about prioritization, fairness, and opportunity costs in resource‐constrained healthcare systems. Any assessment of the real‐world value of medical innovation must therefore address both economic sustainability and social equity.

This exploratory study investigates these challenges by analyzing real‐world survival outcomes and drug‐related costs of modern vs. conventional therapies for r/r DLBCL. While the efficacy of novel agents is well established in trial settings, their real‐world cost‐effectiveness—how survival gains relate to actual treatment costs in routine practice—remains insufficiently characterized, particularly within European healthcare systems. Using patient‐level data from Lower Austria (2018–2022), we constructed multiple analytic cohorts to compare outcomes and calculate ICERs, thereby assessing the value of innovation under real‐world clinical conditions.

## 2 Methods

### 2.1 Study Design

This study was a retrospective real‐world cohort analysis. It included all patients treated for r/r DLBCL in hospitals across Lower Austria between January 1, 2018, and December 31, 2022. Data were extracted from electronic patient records generated during routine clinical care and maintained in the Oncology Information System (OIS) of the Lower Austrian Regional Health Agency (NÖ Landesgesundheitsagentur), Europe's largest regional healthcare provider.

Patients were identified within the OIS by ICD‐10 code C83.3 and by having received more than one line of therapy.

Based on these longitudinal data, cost‐effectiveness analyses were performed, including the calculation of incremental cost‐effectiveness ratios (ICERs) and the construction of an efficiency frontier.

### 2.2 Treatment Regimens

Between 2018 and 2022, the European Medicines Agency (EMA) approved five therapies for r/r DLBCL (www.ema.europa.eu; accessed April 11, 2025). The first approval—polatuzumab vedotin on January 16, 2020—defined the cut‐off used to construct the two calendar‐based cohorts.

### 2.3 Cohort Definitions

To assess both chronological trends in therapy availability and the actual utilization of novel agents, two parallel cohort classifications were applied:

1. Calendar‐based cohorts

Patients were grouped by the start date of second‐line treatment:

- *Calendar cohort 1*: January 1, 2018–December 31, 2019
- *Calendar cohort 2*: January 1, 2020–December 31, 2022

This division reflects the European marketing authorization of polatuzumab vedotin in January 2020, which marked the beginning of broader access to modern therapies. However, because uptake in routine practice may lag behind regulatory approval, patients in calendar cohort 2 were not necessarily treated with modern regimens. The calendar‐based grouping, therefore, reflects treatment availability, but not necessarily treatment utilization.

2 Treatment‐exposure‐based cohorts

Patients were also classified independently of calendar time based on the actual therapies received:

- *Modern therapy cohort*: Received at least one modern therapy at any line of treatment
- *Conventional therapy cohort*: Received only conventional regimens throughout the treatment course

This exposure‐based classification accounts for real‐world crossover and delayed adoption of innovation, allowing a more accurate evaluation of treatment effects and cost‐effectiveness across the entire care pathway.

### 2.4 Therapy, Outcomes, and Statistical Analysis

Detailed information on the study population, therapy, outcome measures, cost evaluation, and the statistical analysis is provided in the Supplementary Methods.

### 2.5 Ethical Considerations

This study was conducted in accordance with the ethical standards of the institutional and national research committees, as well as the 1964 Declaration of Helsinki and its subsequent amendments. Ethical approval was granted by the Ethics Committee of Lower Austria (Reference: GS4‐EK‐4/877–2023). Due to the retrospective design, the Commission for Scientific Integrity and Ethics waived the requirement for informed consent.

## 3 Results

### 3.1 Patient Characteristics (Calendar‐Based Cohorts)

A total of 32 patients with r/r DLBCL were included. Ten patients initiated second‐line therapy during calendar cohort 1 (January 1, 2018–December 31, 2019), and 22 during calendar cohort 2 (January 1, 2020–December 31, 2022). Gender distribution was comparable between cohorts (*p* = 0.811), and median age at the start of second‐line treatment was similar (69 years [range: 46–80] vs. 72 years [range: 58–92]; *p* = 0.190).

Regarding disease status, primary refractory disease was more frequent in cohort 1 (40.0%) than in cohort 2 (18.2%). Relapse within one year of completing first‐line therapy occurred in 40.0% of patients in cohort 1% and 54.5% in cohort 2 (Supplementary Table S2).

### 3.2 Therapies Applied and Response Rates (Calendar‐Based Cohorts)

First‐line treatment regimens were comparable across both cohorts, with most patients receiving R‐CHOP or R‐CHOP–based therapies (such as R‐mini‐CHOP or R‐COMP/R‐mini‐COMP).

The overall response rate (ORR) to second‐line treatment was 50% in calendar cohort 1 (complete response (CR): 40%; partial response (PR): 10%) and 45.5% in calendar cohort 2 (CR: 18.2%; PR: 27.3%) (Supplementary Table S2).

Across both cohorts, approximately half of the patients were eligible for high‐dose chemotherapy followed by autologous stem cell transplantation (ASCT) or CAR‐T cell therapy.

All patients in calendar cohort 1 eventually relapsed or progressed; six received third‐line therapy. In calendar cohort 2, 72.7% of patients progressed after second‐line treatment, and three received third‐line therapy. Two of these later proceeded to fourth‐line treatment, with no clinical benefit observed (progressive disease in both cases) (Supplementary Table S3).

### 3.3 Progression‐Free and Overall Survival (Calendar‐Based Cohorts; no Significant Difference)

**FIGURE 1:**
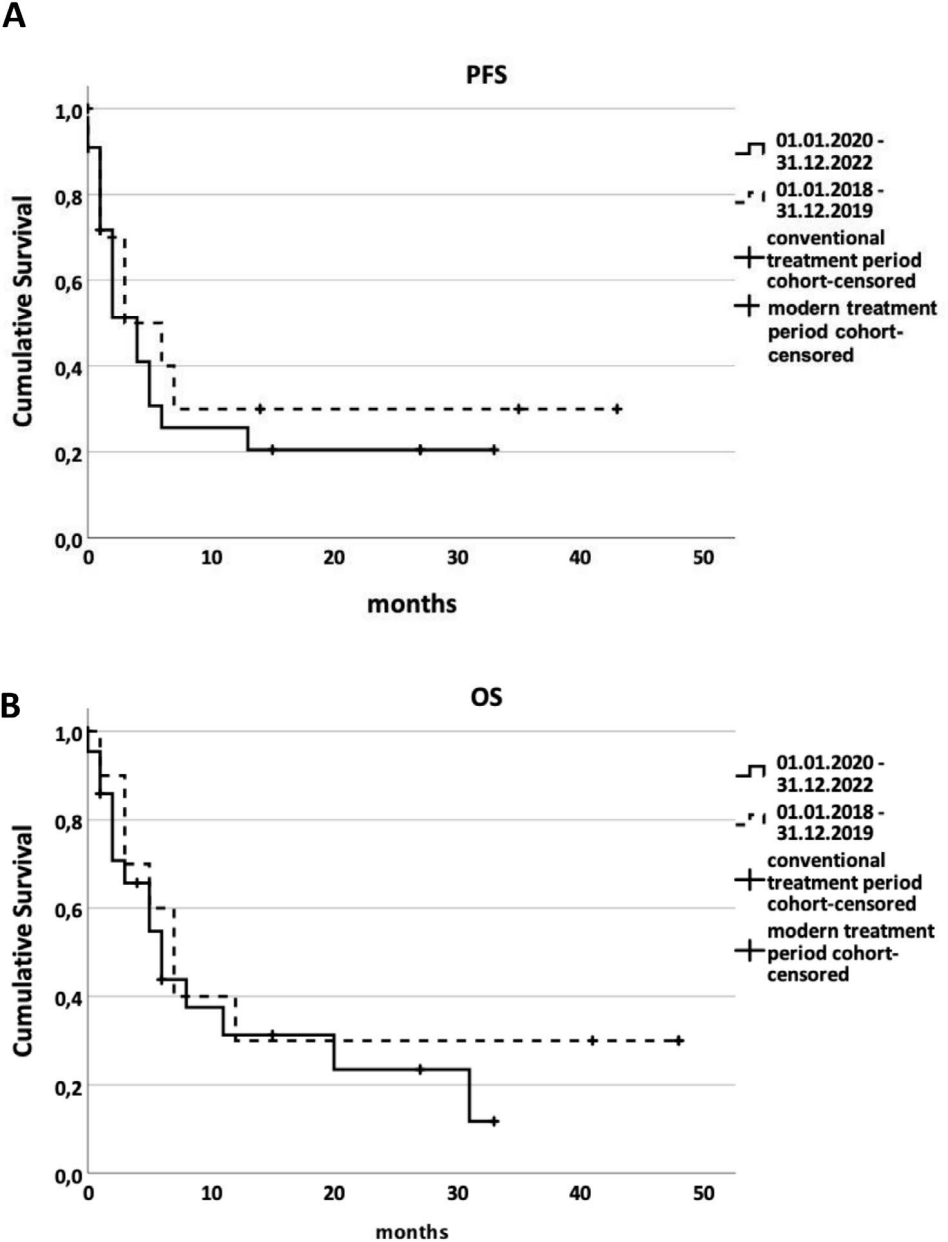
Kaplan–Meier curves showing progression‐free survival (A) and overall survival (B) over time, grouped by calendar period of second‐line treatment initiation.

Remissions were achieved in both calendar‐based cohorts. Median PFS was 3.0 months (95% CI: 0.0–8.2) in cohort 1 and 4.0 months (95% CI: 1.9–6.1) in cohort 2 (*p* = 0.582; Figure 1A). Median OS was 7.0 months (95% CI: 4.0–10.0) in cohort 1 and 6.0 months (95% CI: 2.9–9.1) in cohort 2 (*p* = 0.483; Figure 1B); i.e., PFS and OS did not differ significantly between cohorts.

### 3.4 Patient Characteristics and Survival Outcomes (Treatment‐Exposure Cohorts)

**FIGURE 2:**
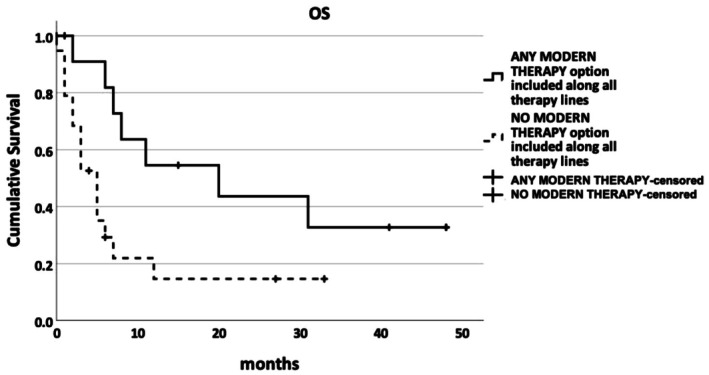
Kaplan–Meier overall survival (OS) curves grouped by exposure to modern therapy at any time during treatment. A significant difference was observed between groups (*p* < 0.05).

In addition, patients were analyzed according to actual therapy exposure. Among all 32 patients, 19 were treated exclusively with conventional regimens (conventional treatment cohort) and 13 received at least one modern therapy at some point (modern treatment cohort). Median OS was significantly longer in the modern treatment cohort (20.0 months; 95% CI: 1.8–38.2) vs. the conventional treatment cohort (5.0 months; 95% CI: 3.0–7.0; *p* = 0.022; Figure 2).

### 3.5 Cost Assessment (Both Cohorts)

Therapy costs were calculated using a standard Austrian patient profile, focusing on direct drug costs (see Materials and Methods and Supplementary Table S4, Table S5). First‐line treatment costs were excluded. ASCT and alloSCT were costed using flat‐rate values.

**Calendar‐based cohorts:**

**FIGURE 3:**
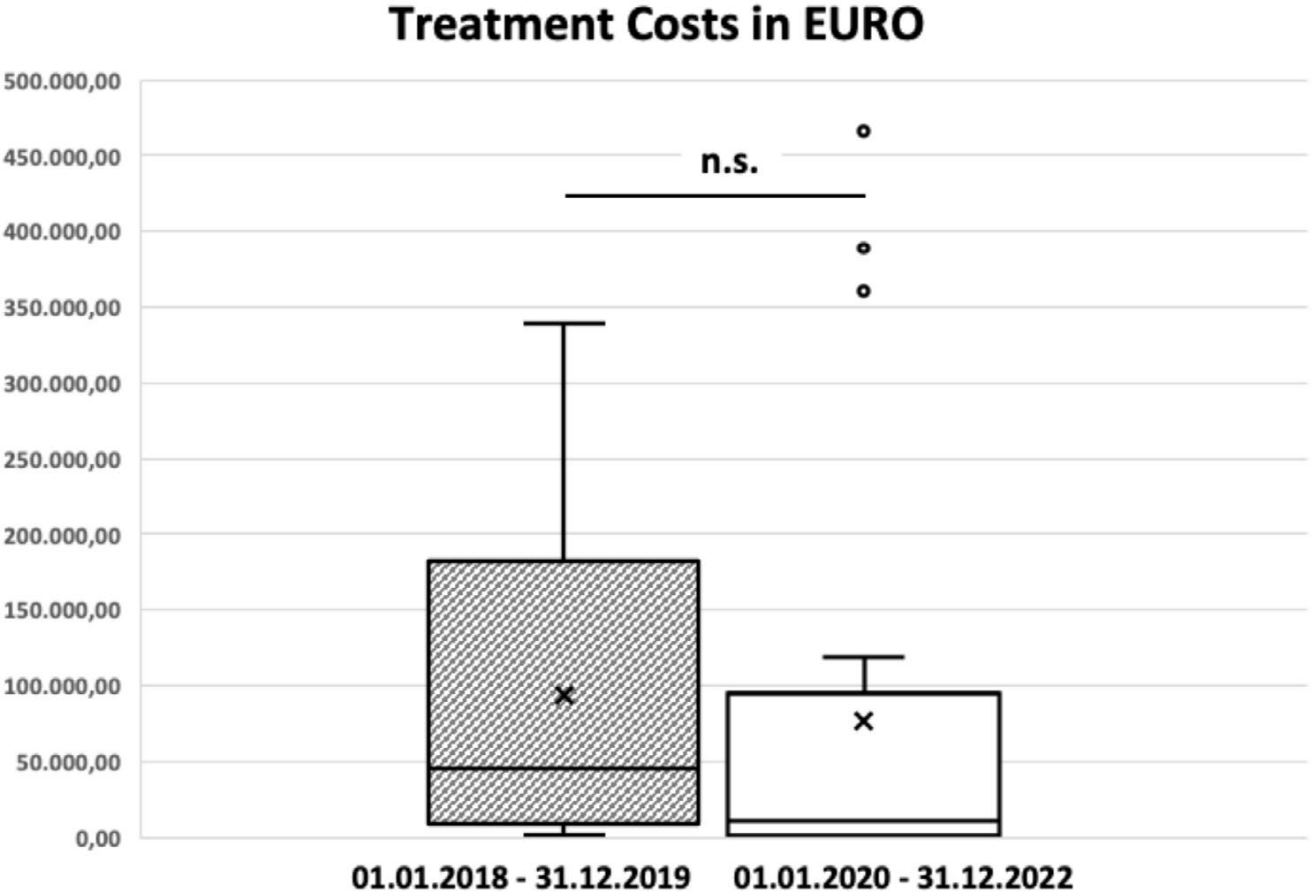
Total treatment costs (€) across all therapy lines for r/r DLBCL, grouped by the date of second‐line therapy initiation. The box plot displays the median, interquartile range (IQR), and 1.5 × IQR whiskers; ×denotes the group means. No significant difference was observed between cohorts (Mann–Whitney U test, *p* = 0.278; n.s.).

- Mean cost in cohort 1: €92,639 (median: €44,710; range: €768–€338,264)
- Mean cost in cohort 2: €76,491 (median: €10,972; range: €535–€465,916)
  There was no significant difference between the two cohorts (*p* = 0.278; Figure 3).

**Treatment‐exposure cohorts:**

- Mean cost in the modern therapy cohort: €178,513 (median: €119,134; range: €19,970–€465,916)
- Mean cost in the conventional therapy cohort: €15,185 (median: €4,359; range: €535–€95,221)

**FIGURE 4:**
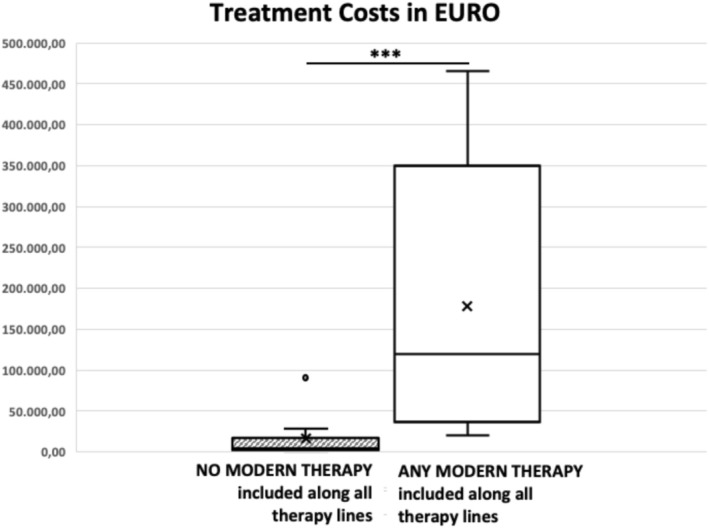
Total treatment costs (€) across all therapy lines for r/r DLBCL, grouped by inclusion of any modern therapy. The box plot displays the median, interquartile range (IQR), and 1.5 × IQR whiskers; × denotes the group means. Differences between groups were highly significant (Mann–Whitney U test, ***p < 0.001).

Costs were significantly higher in patients receiving modern therapies (*p* < 0.001; Figure 4).

### 3.6 ICER and Efficiency Frontier (Treatment‐Exposure Cohorts)

**FIGURE 5:**
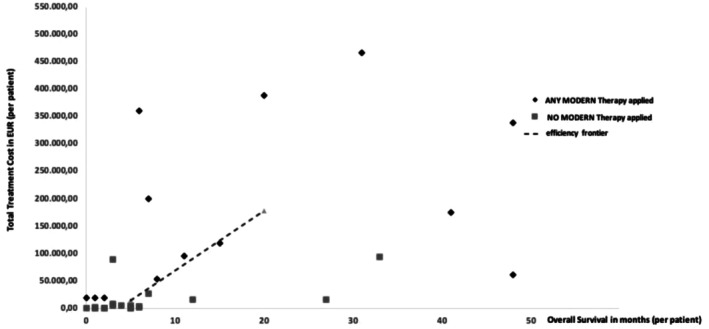
Total treatment cost vs. overall survival (OS) by therapy exposure. Black diamonds represent individual OS and total costs in the modern‐therapy cohort; gray squares represent the conventional cohort. The efficiency frontier (dashed line) connects the conventional‐therapy point (OS = 5 months; cost = €15,185) and the modern‐therapy point (OS = 20 months; cost = €178,513).

To assess cost‐effectiveness, individual total treatment costs were plotted against OS for all patients, grouped by treatment exposure (Figure 5). Each data point represents a patient's cumulative drug‐related costs and observed OS.

For a cohort‐level summary, an efficiency frontier was constructed by linking the median OS and mean total costs of the two treatment‐exposure cohorts:

- Conventional therapy cohort: Median OS = 5 months; mean cost = €15,185
- Modern therapy cohort: Median OS = 20 months; mean cost = €178,513

The resulting incremental cost‐effectiveness ratio (ICER) is:

$$\text{ICER}=\frac{178,513-15,185}{20-5}=\text{€}10,889\;\mathrm{per}\;\text{additional month of}\;\mathrm{OS}$$

This corresponds to €130,662 per life‐year gained.

The efficiency frontier provides a benchmark for comparing the relative value of treatment options. Therapies positioned above the line are less cost‐effective (higher cost per unit of survival gained), whereas those below the line are more cost‐effective.

## 4 Discussion

### 4.1 Limitations of Calendar‐Based Cohorts and Improved Outcomes With Modern Therapies

This retrospective real‐world cohort analysis evaluated treatment patterns and cost‐effectiveness in patients with r/r DLBCL treated in Lower Austria between 2018 and 2022. Comparisons between the two calendar‐based cohorts (2018–2019 vs. 2020–2022) were limited for two main reasons. First, although patients in calendar cohort 1 were treated before the approval of modern therapies, some later received such agents in subsequent treatment lines. Second, and more importantly, modern therapies did not enter routine practice immediately after their approval in early 2020. Many patients in calendar cohort 2, therefore, continued to receive conventional regimens. This lag in adoption may reflect common real‐world barriers, including clinicians' preferences for established regimens, institutional resource constraints, and patient‐level factors such as age, frailty, or comorbidities.

Because of this treatment crossover and delayed uptake, no significant differences in PFS or ORR were observed between the calendar‐based cohorts (Figure 1, Supplementary Table S2). These findings illustrate an important methodological limitation of calendar‐based grouping during periods of therapeutic innovation, when regulatory approval and real‐world utilization do not align. Under such conditions, exposure‐based cohort definitions provide a more accurate representation of actual treatment patterns and their clinical consequences.

Although formal comparisons showed no significant differences, remission durability appeared higher in calendar cohort 2. At the last follow‐up, 27.3% of patients in this cohort remained in remission, compared with none in cohort 1. This pattern is consistent with clinical trial evidence demonstrating durable responses with modern therapies. For example, in the pivotal Phase II study of glofitamab, 78% of patients achieving a complete response remained in remission at 12 months [11]. Likewise, CAR‐T cell therapies have shown long‐term disease control: In ZUMA‐7, median OS for axicabtagene ciloleucel was not reached, and real‐world cohorts have confirmed sustained remissions in a substantial proportion of patients [7, 8, 17, 18].

### 4.2 Clinical Outcomes and Drug Costs by Therapy Exposure

To address the limitations of calendar‐based comparisons, patients were also classified according to their actual treatment exposure. This analysis demonstrated a clear survival advantage for patients who received at least one modern therapy. Median overall survival (OS) was 20 months (95% CI: 1.8–38.2) in the modern therapy cohort compared with 5 months (95% CI: 3.0–7.0) among patients treated exclusively with conventional regimens (*p* = 0.022; Figure 2). Age distributions were similar in the two treatment‐exposure cohorts (median 69 vs. 72 years; *p* = 0.190), making strong age‐related selection bias unlikely. Nevertheless, residual confounding by indication cannot be excluded, as factors such as frailty, comorbidities, and disease biology may have influenced treatment allocation and survival outcomes.

Cost analyses yielded a contrasting picture. When grouped by calendar period, total drug‐related costs did not differ significantly between cohorts. However, when grouped by actual treatment exposure, substantial cost differences emerged. Patients treated with modern therapies incurred markedly higher costs (mean €178,513) than those treated exclusively with conventional regimens (mean €15,185; *p* < 0.001; Figure 4). This finding reflects the high acquisition cost of many novel agents and underscores the importance of evaluating not only clinical outcomes but also the economic implications of real‐world treatment decisions.

### 4.3 Cost‐Effectiveness and ICER Benchmarks

Cost‐effectiveness was evaluated using the treatment‐exposure cohorts to avoid confounding from treatment crossover and delayed implementation of modern therapies. The incremental cost‐effectiveness ratio (ICER) was calculated based on median OS and mean total treatment costs, yielding an ICER of €10,889 per additional life‐month gained (equivalent to €130,662 per life‐year gained). This value corresponds to the slope of the efficiency frontier constructed from the median OS and mean cost of each cohort (Figure 5). The efficiency frontier provides a visual benchmark for comparing the relative value of therapies: Treatments positioned above the line are less cost‐effective, whereas those below are more cost‐effective for a given survival benefit.

Median OS was used to calculate the ICER, consistent with common oncology practice. Pairing median survival with mean costs is methodologically imperfect and may increase uncertainty, but additional approaches—such as sensitivity analyses or stratified sub‐analyses—were not feasible due to sample size constraints. As such, the ICER reported here should be interpreted as an exploratory estimate reflecting real‐world practice rather than a definitive economic evaluation.

The ICER observed in our study is higher than many estimates derived from controlled trial settings but remains within internationally relevant thresholds. For example, Kurte et al. reported an ICER of €28,393 per life‐year gained for tafasitamab‐lenalidomide compared with bendamustine‐rituximab [19], although their evaluation focused on a single line of therapy and was based on trial data rather than real‐world outcomes. Similarly, Choe et al. reported an ICER of USD 99,669 per QALY gained for lisocabtagene maraleucel in the second‐line setting—well within the commonly cited US threshold of USD 150,000 per QALY [20]. Our findings are broadly consistent with these estimates, although direct comparison is limited by the absence of patient‐reported outcomes (PROMs) in our study, which precluded QALY calculations.

Importantly, when Choe et al. incorporated broader societal costs such as productivity loss, the ICER decreased to USD 68,212 per QALY. This reflects the added value of using a societal perspective and integrating PROMs into real‐world evaluations to better capture the full impact of therapy on patients and society. It also underscores the distinction between direct healthcare provider costs and indirect societal costs—two complementary perspectives essential for informed policymaking. In contrast, more conservative analyses based on US list prices have reported substantially higher ICERs, such as USD 684,225 and USD 1,171,909 for axicabtagene ciloleucel and lisocabtagene maraleucel, respectively [21], illustrating the high sensitivity of cost‐effectiveness estimates to drug prices.

### 4.4 National Context: Interpreting Cost‐Effectiveness in Austria

Austria does not have an officially defined cost‐effectiveness threshold for reimbursement decisions, and QALY‐based thresholds are not routinely applied in national health policy [22]. In the absence of a formal benchmark, the WHO recommendation of three times GDP per capita (~€155,484 per QALY for Austria) is often used as a pragmatic proxy [23]. Our estimate of €130,662 per life‐year gained falls below this threshold, suggesting that modern therapies may be considered cost‐effective from an Austrian health system perspective. However, a life‐year gained does not necessarily correspond to one QALY, particularly in r/r DLBCL, where treatment toxicity, symptom burden, and functional decline can impair quality of life. Accordingly, the true cost per QALY is likely higher, although it may still lie within a range deemed acceptable in comparable European healthcare systems.

The scarcity of real‐world cost‐effectiveness studies in Europe reinforces the importance of generating local evidence to inform national health policy. A notable example is the Swedish evaluation by Loftager et al., which extrapolated ZUMA‐7 trial data to estimate an ICER of €50,303 per QALY gained for axicabtagene ciloleucel—well below Sweden's national threshold of €94,077 per QALY [18]. Such international benchmarks can serve as useful reference points for Austria until country‐specific thresholds are formally established. Ultimately, determining acceptable cost‐effectiveness thresholds requires careful consideration of national healthcare priorities, fiscal capacity, and societal preferences regarding equity and access to high‐cost therapies.

### 4.5 Value‐Based Cancer Care and Health Policy Implications

A robust cost‐effectiveness assessment requires integrating both clinical outcomes and patient‐reported quality of life. Patient‐reported outcome measures (PROMs) provide essential insights into patients' physical, emotional, and functional well‐being and are necessary for calculating QALYs [24, 25, 26]. Despite their importance, PROMs remain underutilized in routine oncology care. The European Society for Medical Oncology (ESMO) strongly recommends the systematic collection of PROMs across the cancer care continuum to support evidence‐based decision‐making and high‐quality patient care [27].

Complementary to PROMs, structured clinical benefit frameworks such as the ESMO Magnitude of Clinical Benefit Scale (ESMO‐MCBS) offer validated methods for assessing survival, disease control, and quality of life [28, 29]. Although currently applied mainly to solid tumors, the ESMO‐MCBS is being expanded to hematologic malignancies (https://www.esmo.org/guidelines/esmo‐mcbs/esmo‐mcbs‐for‐solid‐tumours/esmo‐mcbs‐scorecards; https://www.esmo.org/guidelines/esmo‐mcbs/esmo‐mcbs‐for‐haematological‐malignancies; accessed 11.04.2025), reflecting growing recognition of its relevance in lymphoma care. Similar initiatives by the American Society of Clinical Oncology (ASCO) underscore the international trend toward value‐based oncology and the need for transparent, patient‐centered evaluation of new therapies [30].

Our findings corroborate the real‐world clinical effectiveness of modern therapies for r/r DLBCL but also highlight the substantially higher drug costs associated with these treatments. To evaluate their full clinical value, PROMs are urgently needed to quantify the patient experience, capture treatment‐related toxicity, and translate survival outcomes into quality‐adjusted estimates. When combined with clinical benefit frameworks such as the ESMO‐MCBS, PROMs can support more precise and equitable value‐based pricing, reimbursement policies, and resource allocation decisions. These considerations are increasingly important in aging societies with rising cancer incidence and growing demand for high‐cost therapies. Ensuring that access to innovative treatments remains aligned with both clinical benefit and societal priorities will be critical for the long‐term sustainability of healthcare systems.

### 4.6 Strengths and Limitations

A key strength of this study is its comprehensive real‐world perspective. Whereas many previous evaluations of r/r DLBCL rely on clinical trial data and focus on a single line of therapy, our analysis captures the entire treatment trajectory across multiple lines of therapy. This is particularly relevant in r/r DLBCL, where different agents may be used sequentially or in combination, and where cumulative treatment effects and costs are highly variable. Assessing outcomes and total treatment costs across the full care pathway—an important contribution of this study—is seldom undertaken in the existing literature.

Another strength is the dataset's real‐world nature. Clinical trials usually include highly selected patient populations due to strict eligibility criteria, whereas routine clinical care involves patients with a wider range of ages, frailty, and comorbidities. Therefore, the findings presented here reflect the full heterogeneity of real‐world patients and provide valuable insights that complement those obtained from clinical trials.

The data originate from a single integrated health system that covers an entire federal state. The Lower Austrian Regional Health Agency is the largest healthcare operator in Europe, with more than 28,000 employees, 27 hospitals, and over 50 care facilities (www.landesgesundheitsagentur.at; accessed 11.04.2025). This centralized structure ensures uniform documentation across the entire network. The Oncology Information System (OIS) undergoes systematic quality control, containing over 100,000 patient records with quarterly audits of approximately 6500 entries. Prior publications confirm the OIS as a robust data source [31, 32].

Key limitations include the small sample size inherent in r/r DLBCL research, due to the low incidence and high cure rate (60%) after first‐line therapy. Therefore, this analysis should be considered exploratory. Future studies should aim to integrate data from Lower Austria with information from other regions or the entire country to increase statistical power. Another key limitation is the absence of PROMs, which hinders the calculation of QALYs and limits the interpretation of cost‐effectiveness results relative to international thresholds and health economic benchmarks. Further, the retrospective design introduces risks of selection bias and unmeasured confounding. Patients treated outside Lower Austria or in private hospitals were not captured, potentially underrepresenting certain demographic groups—a common issue in retrospective studies, underscoring the importance of carefully interpreting results and validating findings through prospective trial‐based research. Together, these constraints highlight the need for a nationwide, harmonized electronic clinical documentation system.

Finally, studies of this kind are only feasible in healthcare systems that guarantee universal access to high‐cost therapies. Although some countries may offer earlier access, such as the United States, availability often depends on insurance coverage. Austria's universal healthcare system ensures that treatment decisions are based on clinical need rather than financial capacity—an important condition for generating unbiased real‐world evidence.

## 5 Conclusion

Modern therapies for r/r DLBCL provide significant survival benefits in routine care and demonstrate acceptable cost‐effectiveness. Although treatment costs are substantially higher, the ICER observed in this real‐world Austrian cohort falls within internationally recognized thresholds, supporting the health‐economic value of these therapies.

A key methodological finding of this study is the delay in the uptake of innovative agents after EMA approval. This gap between regulatory authorization and real‐world utilization limits the interpretability of calendar‐based cohort analyses and should be carefully considered in future health services research and policy evaluation.

The absence of PROMs prevented QALY estimation, underscoring the need for systematic PROM collection and the integration of standardized benefit frameworks—such as the ESMO‐MCBS—into routine clinical practice. These tools are essential for comprehensive, patient‐centered value assessment.

Austria currently lacks a formal QALY‐based cost‐effectiveness threshold. Our findings highlight the importance of developing national benchmarks that reflect healthcare priorities, fiscal constraints, and societal values. Until such thresholds are defined, international guidance—including the WHO's 3 × GDP per capita benchmark—and thresholds from comparable health systems offer a pragmatic interim reference.

This exploratory study was made possible through access to a unified oncology information system (OIS) covering the entire public hospital network of Lower Austria—an uncommon degree of data integration in Austria's otherwise fragmented documentation landscape. Establishing interoperable nationwide standards is essential to enable larger‐scale, more robust evaluations of care quality, outcomes, and cost‐effectiveness.

Looking ahead, health policy will need to balance therapeutic innovation with system sustainability to ensure equitable access to high‐value cancer therapies—particularly in aging societies facing increasing demand and constrained public budgets.

## Author Contributions

**Josef Singer:** conceptualization, investigation, writing – original draft, methodology, validation, visualization, writing – review and editing, formal analysis, data curation. **Sandra Gottsauner‐Wolf:** investigation, data curation, writing – review and editing. **Doris A. Behrens:** conceptualization, investigation, data curation, supervision, writing – review and editing.

## Funding

This study was supported by this research did not receive any specific funding from public, commercial, or non‐profit organizations.

## Disclosure

During the preparation of this work, the authors used ChatGPT to shorten and streamline the manuscript. After employing this tool, they reviewed and edited the content as necessary and took full responsibility for the publication's content.

## Conflicts of Interest

J.S. has received honoraria from AbbVie, Amgen, Angelini, Gilead, Janssen, Kite, Merck, Merck Sharp & Dohme, Miltenyi, Novartis, Pfizer, Roche, and Servier for speaking engagements or consultancy. The other authors declare no conflicts of interest.

## Supporting information


**Data S1:** Supporting Information.

## Data Availability

The data that support the findings of this study are available on request from the corresponding author. The data are not publicly available due to privacy or ethical restrictions.
