# Supplementary material for: Retrospective Cohort Analysis of Treatment Patterns, Survival, and Cost‐Effectiveness in Relapsed/Refractory Diffuse Large B‐Cell Lymphoma in Lower Austria (2018–2022)
Source: Cancer Med. 2026 Feb 24;15(3):e71667. doi: 10.1002/cam4.71667 (PMC12932070; doi:10.1002/cam4.71667)
Supplement: Supplementary file 1 — Data S1: Supporting Information. [file CAM4-15-e71667-s001.docx]

**Supplementary Information**

**Supplementary Methods**

**Study Population**

Eligible patients were adults (≥18 years) with a confirmed diagnosis of r/r DLBCL who received treatment at a hospital in Lower Austria within the defined period. Patients referred for advanced therapies such as ASCT at tertiary centres in other Austrian states were included if their primary diagnosis and initial treatment occurred in Lower Austria.

Patients were excluded if they were treated outside the study period, had a haematological malignancy other than r/r DLBCL, were under 18 years of age, or received only best supportive care due to frailty.

**Treatment Regimens**

Between 2018 and 2022, five therapies received marketing authorisation from the European Medicines Agency (EMA) for the treatment of r/r DLBCL ([www.ema.europa.eu](http://www.ema.europa.eu); accessed 11 April 2025). The first of these, polatuzumab-vedotin, was approved on 16 January 2020, which served as the cut-off point for defining two calendar-based cohorts in this study.

The five EMA-approved modern therapies included in the analysis are polatuzumab-vedotin, tafasitamab, axicabtagene ciloleucel, lisocabtagene maraleucel and glofitamab (see Supplementary Table S1).

Conventional regimens used prior to this shift included salvage chemotherapy protocols such as R-DHAP (rituximab, dexamethasone, high-dose cytarabine, cisplatin), R-ICE (rituximab, ifosfamide, carboplatin, etoposide), and others, often followed by ASCT in eligible patients.

**Outcome Measures**

Clinical outcomes included overall response rate (ORR), progression-free survival (PFS), and overall survival (OS). ORR was based on clinical or radiological response as documented in the OIS. PFS was calculated from the date of second-line therapy initiation to disease progression or death, whichever occurred first. OS was defined as the time from second-line therapy initiation to death from any cause. Patients without an event were censored at the date of last follow-up.

**Cost Evaluation**

For each patient, treatment costs were calculated by multiplying the estimated cost of one treatment cycle—based on a standard Austrian patient profile (170 cm, 75 kg, 1.87 m² body surface area)—by the actual number of cycles administered. If a patient received more than one line of therapy in the r/r setting, costs were summed across all applicable treatment lines. First-line treatment costs were excluded from the analysis, as the focus was exclusively on therapies used after relapse or treatment failure.

Only direct drug acquisition costs were considered. Costs related to co-medication, diagnostics, hospitalisation, and toxicity management were excluded. Prices were based on Austrian reimbursement tariffs, when available, or ex-factory prices.

For ASCT and allogeneic stem cell transplantation (alloSCT), drug costs could not be isolated and were instead approximated using flat-rate estimates—EUR 93,500 and EUR 175,000, respectively, based on publicly available tariffs from Charité University Hospital, Berlin (https://www.charite.de/fileadmin/user_upload/portal/klinikum/behandlung_stationaer/Entgelttarif.pdf; accessed 20 September 2024).

**Statistical Analysis**

Descriptive statistics were used to summarise patient characteristics, treatment patterns, outcomes, and treatment costs. Outcomes were analysed for both cohort types: period-based and treatment-based.

Kaplan-Meier curves were plotted for PFS and OS, with between-group comparisons conducted using the log-rank test. Categorical variables were analysed using the chi-squared test, and non-parametric group comparisons using the Mann-Whitney U test. A p-value < 0.05 (two-tailed) was considered statistically significant.

All statistical analyses were performed using Microsoft Excel for Microsoft 365 (Version 2303) and IBM SPSS Statistics for macOS (Version 29, IBM Corp., Armonk, NY, USA).

**Supplementary Table S1. Overview of Modern Therapies for r/r DLBCL**

The past decade has seen major therapeutic advances in the treatment of relapsed or refractory diffuse large B-cell lymphoma (r/r DLBCL), particularly with the approval of targeted immunotherapies. Supplementary Table S1 provides an overview of the most relevant modern therapies approved by the European Medicines Agency (EMA) between 2020 and 2023. These include:

- **Antibody-drug conjugates (ADCs)**, such as *polatuzumab-vedotin*, which combine monoclonal antibody specificity with a cytotoxic payload [1,2].
- **Fc-engineered monoclonal antibodies**, such as *tafasitamab*, designed for enhanced immune effector function [3–5].
- **Chimeric antigen receptor (CAR) T-cell** therapies, which genetically reprogram patient-derived T-cells to target CD19-expressing lymphoma cells [6–10].
- **Bispecific antibodies**, such as *epcoritamab* and *glofitamab*, which simultaneously bind CD3+ T-cells and B-cell antigens (CD20), redirecting cytotoxicity to tumour cells [11–13].

These therapies have shown promising response rates and, in some cases, durable remissions—even in heavily pretreated or transplantation-ineligible patients. However, their clinical application varies in terms of logistics, toxicity profiles, and cost. Table 1 summarises key trial data, including molecular targets, overall survival (OS), overall and complete response rates (ORR/CR), and pivotal clinical trials. These data are derived from controlled trial settings and may differ from outcomes observed in routine clinical care, as discussed in the main manuscript.

| Therapy Type | Drug  (Trade Name) | Target | Trial Name / Phase | Median OS (months) | ORR / CR (%) |
| --- | --- | --- | --- | --- | --- |
| Antibody-Drug Conjugate | Polatuzumab-vedotin (Polivy®) | CD79b | NCT02257567 / Phase II | 12.4 (Pola-BR) vs 4.7 (BR) | 41.5 / 38.7 |
| Fc-Enhanced Anti-CD19 Antibody | Tafasitamab (Minjuvi®) + lenalidomide | CD19 | L-MIND / Phase II | 33.5 | 57.5 / 41.3 |
| CAR T-Cell Therapy | Axicabtagene ciloleucel (Yescarta®) | CD19 | ZUMA-1 / Phase II, ZUMA-7 / Phase III | NR (ZUMA-1);  8.3 (ZUMA-7, EFS) | 82 / 54 (ZUMA-1);  83 / 65 (ZUMA-7) |
| CAR T-Cell Therapy | Lisocabtagene maraleucel (Breyanzi®) | CD19 | TRANSCEND / Phase II, PILOT / Phase II | NR (preliminary) | 73 / 53 |
| CAR T-Cell Therapy | Tisagen-lecleucel (Kymriah®) | CD19 | JULIET / Phase II | NR (JULIET) | 52 / 40 |
| Bispecific Antibody | Epcoritamab (Tepkinly®) | CD20 & CD3 | EPCORE NHL-1 / Phase I-II | 44.6% at 24 months | 63 / 40 |
| Bispecific Antibody | Glofitamab (Columvi®) | CD20 & CD3 | NP30179 /  Phase I-II | 12-month PFS: 37% | 39 (CR only reported) |

***Table S1. Overview of Modern Therapies for r/r DLBCL***

*NR = Not Reached; OS = Overall Survival; PFS = Progression-Free Survival*

***References:***

1. Sehn LH, Herrera AF, Flowers CR et al. Polatuzumab Vedotin in Relapsed or Refractory Diffuse Large B-Cell Lymphoma. J Clin Oncol 2020; 38: 155-165.

2. Sehn LH, Hertzberg M, Opat S et al. Polatuzumab vedotin plus bendamustine and rituximab in relapsed/refractory DLBCL: survival update and new extension cohort data. Blood Adv 2022; 6: 533-543.

3. Hoy SM. Tafasitamab: First Approval. Drugs 2020; 80: 1731-1737.

4. Salles G, Duell J, Gonzalez Barca E et al. Tafasitamab plus lenalidomide in relapsed or refractory diffuse large B-cell lymphoma (L-MIND): a multicentre, prospective, single-arm, phase 2 study. Lancet Oncol 2020; 21: 978-988.

5. Duell J, Abrisqueta P, Andre M et al. Tafasitamab for patients with relapsed or refractory diffuse large B-cell lymphoma: final 5-year efficacy and safety findings in the phase II L-MIND study. Haematologica 2024; 109: 553-566.

6. Mitra A, Barua A, Huang L et al. From bench to bedside: the history and progress of CAR T cell therapy. Front Immunol 2023; 14: 1188049.

7. Neelapu SS, Locke FL, Bartlett NL et al. Axicabtagene Ciloleucel CAR T-Cell Therapy in Refractory Large B-Cell Lymphoma. N Engl J Med 2017; 377: 2531-2544.

8. Locke FL, Miklos DB, Jacobson CA et al. Axicabtagene Ciloleucel as Second-Line Therapy for Large B-Cell Lymphoma. N Engl J Med 2022; 386: 640-654.

9. Abramson JS, Palomba ML, Gordon LI et al. Lisocabtagene maraleucel for patients with relapsed or refractory large B-cell lymphomas (TRANSCEND NHL 001): a multicentre seamless design study. Lancet 2020; 396: 839-852.

10. Schuster SJ, Bishop MR, Tam CS et al. Tisagenlecleucel in Adult Relapsed or Refractory Diffuse Large B-Cell Lymphoma. N Engl J Med 2019; 380: 45-56.

11. Trabolsi A, Arumov A, Schatz JH. T Cell-Activating Bispecific Antibodies in Cancer Therapy. J Immunol 2019; 203: 585-592.

12. Thieblemont C, Karimi YH, Ghesquieres H et al. Epcoritamab in relapsed/refractory large B-cell lymphoma: 2-year follow-up from the pivotal EPCORE NHL-1 trial. Leukemia 2024.

13. Dickinson MJ, Carlo-Stella C, Morschhauser F et al. Glofitamab for Relapsed or Refractory Diffuse Large B-Cell Lymphoma. N Engl J Med 2022; 387: 2220-2231.

**Supplementary Table S2. Patient characteristics and detailed information on applied first- and second-line therapy.**

|  | **Conventional Treatment Period**  **(01.01.2018 – 31.12.2019)** | **Modern Treatment Period**  **(01.01.2020 – 31.12.2022)** |
| --- | --- | --- |
| n | 10 | 22 |
| Gender, n (% of all patients):  female  male | 5 (50)  5 (50) | 10 (45.5)  12 (54.5) |
| Age at 2^nd^ line therapy start  in years (mean; min – max) | 69 (46 – 80) | 73 (58 – 92) |
| Disease state: n (% of all patients)  Primary refractory disease  Relapse within 1 year  Late relapse | 4 (40)  4 (40)  2 (20) | 4 (18.2)  12 (54.5)  6 (27.3) |
| Response to 1^st^ line therapy:  n (% of all patients)  CR  PR  SD  PD  ORR | 3 (30)  3 (30)  1 (10)  3 (30)  60% | 7 (31.8)  11 (50)  2 (9.1)  2 (9.1)  81.8% |
| Applied 1^st^ line therapy:  n (% of all patients)  R-CHOP  R-COMP  R-mini-CHOP  R-mini-COMP  BR  DA-R-EPOCH  MATRix | 5 (50)  4 (40)  0 (0)  0 (0)  0 (0)  1 (10)  0 (0) | 7 (31.8)  6 (27.3)  3 (13.6)  2 (9.1)  3 (13.6)  0  1 (4.5) |
| Response 2^nd^ line therapy:  n (% of all patients)  CR  PR  SD  PD  **ORR** | 4 (40)  1 (10)  1 (10)  4 (40)  **50%** | 4 (18.2)  6 (27.3)  1 (4.5)  11 (50)  **45.5%** |
| Applied 2^nd^ line therapy:  n (% of all patients)    **Curative intention:**  R-DHAP  R-DHAP + BEAM + ASCT  O-DHAP + BEAM + ASCT  R-ICE  R-ICE + BEAM + ASCT  HD-MTX  MATRix  MATRix + BEAM + ASCT  O-GDP + allogeneic SCT  Pola-BR  Tafa-Len  Ibrutinib + CAR-T cells  (Lisocabtagene Maraleucel)  Tisagenlecleucel  **Palliative intention**  R-Benda  R-CHOP  R-COMP  R-mini-COMP  R-EPOCH  R-GDCarbo  R-GemOx  R^2^ (Rituximab-Lenalidomide) | **5 (50)**  1 (10)  0 (0)  0 (0)  1 (10)  0 (0)  1 (10)  0 (0)  1 (10)  1 (10)  0 (0)  0 (0)  0 (0)  0 (0)  **5 (50)**  0 (0)  0 (0)  0 (0)  0 (0)  1 (10)  1 (10)  0 (0)  3 (30) | **11 (50)**  0 (0)  1 (4.5)  1 (4.5)  0 (0)  2 (9.1)  0 (0)  1 (4.5)  0 (0)  0 (0)  3 (13.6)  1 (4.5)  1 (4.5)  1 (4.5)  **11 (50)**  2 (9.1)  1 (4.5)  1 (4.5)  2 (9.1)  0 (0=  0 (0)  5 (22.7)  0 (0) |
| **Progress/Relapse after 2^nd^ line therapy:** | **10/10 (100%)** | **16/22 (72.7%)** |

**Supplementary Table S3. Detailed information on applied further lines of therapy after failure of second-line treatment.**

|  | **Conventional Treatment Period**  **(01.01.2018 – 31.12.2019)** | **Modern Treatment Period**  **(01.01.2020 – 31.12.2022)** |
| --- | --- | --- |
| 3^rd^ line therapy:  n (% of all progressed  2^nd^ line patients)  Yes  No | 6 (60)  4 (40) | 3 (18.7)  13 (81.3) |
| Response 3^rd^ line therapy:  n (% of all 3^rd^ line patients)  CR  PR  SD  PD  **ORR** | 3 (50)  0 (0)  0 (0)  3 (50)  **50%** | 1 (33.3)  0 (0)  0 (0)  2 (66.7)  **33.3%** |
| Applied 3^rd^ line-therapy:  n (% of all 3^rd^ line patients)  R-COMP  Pola-BR  Pola-BR + alloSCT  R-GemVin  Ibrutinib  R^2^ (Rituximab-Lenalidomide) | 1 (16.7)  1 (16.7)  1 (16.7)  1 (16.7)  1 (16.7)  0 (0) | 0 (0)  1 (33.3)  0 (0)  0 (0)  0 (0)  2 (66.6) |
| **Progress after 3^nd^ line therapy:** | **3/6 (50%)** | **3/3 (100%)** |
| 4^th^ line therapy:  n (% of all progressed  3^rd^ line patients)  Yes  No | 0 (0)  3 (100) | 2 (66.7)  1 (33.3) |
| Response 4^th^ line therapy:  n (% of all 4^th^ line patients)  CR  PR  SD  PD  **ORR** | n.a.  n.a.  n.a.  n.a.  **n.a.** | 0 (0)  0 (0)  0 (0)  2 (100)  **0%** |
| Applied 4^th^ line therapy:  n (% of all 4^th^ line patients)  Pola-BR  Tafa - Len | n.a.  n.a. | 1 (50)  1 (50) |
| **Progress after 4^th^ line therapy:** | **n.a.** | **2/2 (100%)** |

**Supplementary Table S4. Therapy costs of conventional therapies for one treatment cycle normalized to a standard patient (170 cm, 75 kg, 1.87m^2^ body surface area).**

| **Protocol** | **Dosage/Unit** | **Price/Unit (Euro)** | **Dosage applied** | **Costs/Cycle (Euro)** |
| --- | --- | --- | --- | --- |
| ***Immuno-Chemotherapy*** | | | | |
| ***BR*** *(bendamustine, rituximab)  - 1 cycle á 28 days, 6 cycles intended* | | | | |
| rituximab | 500mg | 201.48 | Day 1: 375 mg/m^2^ | 402.96 |
| bendamustine | 25mg | 10 | Day 1+2: 90mg/m^2^ | 132.20 |
| *Total costs for 1 cycle* | | | | **535.16** |
| ***HD-MTX*** *(high-dose methotrexate)  - 1 cycle á 21 days, 3 cycles intended* | | | | |
| methotrexate | 1000mg | 110.95 | Day 1: 4000 mg/m^2^ | 829.91 |
| *Total costs for 1 cycle* | | | | **829.91** |
| ***MATRix*** *(methotrexate, cytarabine, thiotepa, rituximab)  - 1 cycle á 21 days, 4 cycles intended* | | | | |
| rituximab | 500mg | 201.48 | Day 1+6: 375 mg/m^2^ | 805.92 |
| methotrexate | 1000mg | 110.95 | Day 7: 3500mg/m^2^ | 726.17 |
| cytarabine | 2000mg | 21.95 | 2x/12 h Day 8+9: 2.000 mg/m^2^ | 87.80 |
| thiotepa | 15mg | 225.50 | Day 10: 30mg/m^2^ | 843.37 |
| *Total costs for 1 cycle* | | | | **2463.26** |
| ***R-CHOP*** *(rituximab, cyclophosphamide, doxorubicin, vincristine, prednisone)  - 1 cycle á 21 days, 6 cycles intended* | | | | |
| rituximab | 500mg | 201.48 | Day 1: 375 mg/m^2^ | 402.96 |
| cyclophosphamide | 1000mg | 25.88 | Day 1: 750 mg/m^2^ | 51.76 |
| doxorubicin | 50mg | 124.65 | Day 1: 50 mg/m^2^ | 233.10 |
| vincristine | 2mg | 28.45 | Day 1: 2 mg | 28.45 |
| prednisone | 25mg pills, 1 package á 40 pieces | 20.55 | Day 1-5: 100 mg | 10.30 |
| *Total costs for 1 cycle* | | | | **726.57** |
| ***R-COMP*** *(rituximab, cyclophosphamide, liposomal doxorubicin, vincristine, prednisone)  - 1 cycle á 21 days, 6 cycles intended* | | | | |
| rituximab | 500mg | 201.48 | Day 1: 375 mg/m^2^ | 402.96 |
| cyclophosphamide | 1000mg | 25.88 | Day 1: 750 mg/m^2^ | 51.76 |
| liposomal doxorubicin | 100mg | 2405.85 | Day 1: 50 mg/m^2^ | 2249.47 |
| vincristine | 2mg | 28.45 | Day 1: 2 mg | 28.45 |
| prednisone | 25mg pills, 1 package á 40 pieces | 20.55 | Day 1-5: 100 mg | 10.30 |
| *Total costs for 1 cycle* | | | | **2742.94** |
| ***R-DHAP*** *(rituximab, dexamethasone, high-dose cytarabine, cisplatin)  - 1 cycle á 21 days, 3 cycles intended* | | | | |
| rituximab | 500mg | 201.48 | Day 1: 375 mg/m^2^ | 402.96 |
| dexamethasone | 40mg | 13.95 | Day 1-4: 40 mg | 55.80 |
| cytarabine | 2000mg | 21.95 | 2x/12 h Day 2: 2.000 mg/m^2^ | 87.80 |
| cisplatin | 100mg | 13.48 | Tag 1: 100 mg/m2 | 26.96 |
| *Total costs for 1 cycle* | | | | **573.52** |
| ***R-EPOCH*** *(rituximab, etoposide, cyclophosphamide, doxorubicin, vincristine, prednisone)  - 1 cycle á 21 days, 6 cycles intended* | | | | |
| rituximab | 500mg | 201.48 | Day 1: 375 mg/m^2^ | 402.96 |
| etoposide | 200mg | 16.50 | Day 1-5: 50 mg/m^2^ | 38.57 |
| cyclophosphamide | 1000mg | 25.88 | Day 5: 750 mg/m^2^ | 51.76 |
| doxorubicin | 50mg | 124.65 | Day 1-5: 10 mg/m^2^ | 233.10 |
| vincristine | 2mg | 28.45 | Day 1-5: 0.4 mg | 28.45 |
| prednisone | 25mg pills, 1 package á 40 pieces | 20.55 | Day 1-5: 60 mg/m^2^ | 12.84 |
| *Total costs for 1 cycle* | | | | **767.68** |
| ***R-GDCarbo*** *(rituximab, gemcitabine, carboplatin)  - 1 cycle á 21 days, 6 cycles intended* | | | | |
| rituximab | 500mg | 201.48 | Day 1: 375 mg/m^2^ | 402.96 |
| gemcitabine | 2000mg | 14.88 | Day 1+8: 1000 mg/m^2^ | 29.76 |
| dexamethasone | 40mg | 13.95 | Day 1-4: 40 mg | 55.80 |
| carboplatin | 450mg | 22.00 | Day 1: AUC 5 | 44.00 |
| *Total costs for 1 cycle* | | | | **532.52** |
| ***R-GemOx*** *(rituximab, gemcitabine, oxaliplatin)  - 1 cycle á 14 days, 6 cycles intended* | | | | |
| rituximab | 500mg | 201.48 | Day 1: 375 mg/m^2^ | 402.96 |
| gemcitabine | 2000mg | 14.88 | Day 2: 1000 mg/m^2^ | 14.88 |
| oxaliplatin | 200mg | 18.21 | Day 2: 100 mg/m^2^ | 18.21 |
| *Total costs for 1 cycle* | | | | **436.05** |
| ***R-GemVin*** *(rituximab, gemcitabine, vinorelbine)  - 1 cycle á 21 days, 6 cycles intended* | | | | |
| rituximab | 500mg | 201.48 | Day 1: 375 mg/m^2^ | 402.96 |
| gemcitabine | 2000mg | 14.88 | Day 1+8: 1000 mg/m^2^ | 29.76 |
| vinorelbine | 10mg | 246.40 | Day 1+8: 25 mg/m^2^ | 2303.84 |
| *Total costs for 1 cycle* | | | | **2736.56** |
| ***R-ICE*** *(rituximab, ifosfamide, carboplatin, etoposide)  - 1 cycle á 21 days, 3 cycles intended* | | | | |
| rituximab | 500mg | 201.48 | Day 1: 375 mg/m^2^ | 402.96 |
| ifosfamide | 2000mg | 67.88 | Day 2: 5000 mg/m^2^ | 339.40 |
| mesna | 400mg | 2.47 | Day 2: 5000 mg/m^2^ | 59.21 |
| carboplatin | 450mg | 22.00 | Day 2: AUC 5 | 44.00 |
| etoposide | 200mg | 16.50 | Day 1-3: 100 mg/m^2^ | 49.50 |
| *Total costs for 1 cycle* | | | | **895.07** |
| ***Targeted Therapy*** | | | | |
| ***Ibrutinib***  *- 1 cycle á 28 days, taken until progression* | | | | |
| ibrutinib | 560mg pills,  1 package á 28 pieces | 4894.26 | 560mg/day | 4894.26 |
| *Total costs for 1 cycle* | | | | **4894.26** |

| ***“Immune Only”*** | | | | |
| --- | --- | --- | --- | --- |
| ***R^2^*** *(rituximab, lenalidomide)* ***– before March 2022***  *- 1 cycle á 28 days, intended until progression* | | | | |
| rituximab | 500mg | 201.48 | Day 1: 375 mg/m^2^ | 402.96 |
| lenalidomide | 25mg pills, 1 package á 21 pieces | 4228.31 | Day 1-21: 25mg | 4228.31 |
| *Total costs for 1 cycle* | | | | **4631.27** |
| ***R^2^*** *(rituximab, lenalidomide)* ***– after March 2022***  *- 1 cycle á 28 days, intended until progression* | | | | |
| rituximab | 500mg | 201.48 | Day 1: 375 mg/m^2^ | 402.96 |
| lenalidomide | 25mg pills, 1 package á 21 pieces | 611.60 | Day 1-21: 25mg | 611.60 |
| *Total costs for 1 cycle* | | | | **1014.56** |
| ***Cellular Therapies*** | | | | |
| ***ASCT*** | | | | |
| BEAM |  |  |  |  |
| Carmustin (BCNU) | 100mg | 1392.55 | Day -7: 300mg/m^2^ | 7812.21 |
| cytarabine | 2000mg | 21.95 | 2x/12 h Day -6 to -3: 200 mg/m^2^ | 23.84 |
| etoposide | 200mg | 16.50 | Day -6 to -3: 200mg/m^2^ | 123.42 |
| melphalan | 50mg | 250.55 | Day -2: 140mg/m^2^ | 1311.88 |
| autologous stem cell transplantation + supportive treatment | Xx |  | Day 0:  2x10^6^ CD34+ cells/kg |  |
| *Total costs (flat charge)* | | | | **93500** |
| ***AlloSCT*** | | | | |
| Flu/Cy |  |  |  |  |
| Fludarabine | 250mg | 1638.00 | Day -6 to -2: 30mg/m^2^ | 1837.84 |
| cyclophosphamide | 1000mg | 25.88 | Day -6 to -2: 500mg/m^2^ | 120.99 |
| allogeneic stem cell transplantation + supportive treatment |  |  | Day 0:  2x10^6^ CD34+ cells/kg |  |
| *Total costs (flat charge)* | | | | **175000** |

**Supplementary Table S5. Therapy costs of modern therapies for one treatment cycle normalized to a standard patient (170 cm, 75 kg, 1.87m^2^ body surface area).**

| **Protocol** | **Dosage/Unit** | **Price/Unit (Euro)** | **Dosage applied** | **Costs/ Cycle (Euro)** |
| --- | --- | --- | --- | --- |
| ***Immuno-Chemotherapy*** | | | | |
| ***O-DHAP*** *(obinutuzumab, dexamethasone, high-dose cytarabine, cisplatin)  - 1 cycle á 21 days, 3 cycles intended* | | | | |
| obinutuzumab | 1000mg | 5024.40 | Cycle 1:  100mg at day 1,  900mg at day 2, 1000mg at days 8 & 15 | 15073.20 |
|  |  |  | Cycle 2 onwards:  1000mg at day 1 | 5024.40 |
| dexamethasone | 40mg | 13.95 | Day 1-4: 40 mg | 55.80 |
| cytarabine | 2000mg | 21.95 | 2x/12 h Day 2:  2.000 mg/m^2^ | 87.80 |
| cisplatin | 100mg | 13.48 | Day 1: 100 mg/m2 | 26.96 |
| *Total costs for cycle 1* | | | | **15243.76** |
| *Total costs for cycle 2 onwards* | | | | **5194.96** |
| ***O-GDP*** *(obinutuzumab, gemcitabine, dexamethasone, cisplatin)  - 1 cycle á 21 days, 3 cycles intended* | | | | |
| obinutuzumab | 1000mg | 5024.40 | Cycle 1:  100mg at day 1,  900mg at day 2, 1000mg at days 8 & 15 | 15073.20 |
|  |  |  | Cycle 2 onwards:  1000mg at day 1 | 5024.40 |
| gemcitabine | 2000mg | 14.88 | Day 1+8: 1000 mg/m^2^ | 29.76 |
| dexamethasone | 40mg | 13.95 | Day 1-4: 40 mg | 55.80 |
| cisplatin | 100mg | 13.48 | Day 1: 75 mg/m^2^ | 18.91 |
| *Total costs for cycle 1* | | | | **15177.67** |
| *Total costs for cycle 2 onwards* | | | | **5128.87** |
| ***Pola-BR*** *(polatuzumab-vedotin, bendamustine, rituximab)  - 1 cycle á 21 days, 6 cycles intended* | | | | |
|  |  |  |  |  |
| bendamustine | 25mg | 10 | Day 1+2: 90mg/m^2^ | 132,20 |
| Polatuzumab- vedotin | 140mg | 9800.00 | Day 1: 1.8mg/kg | 9450 |
| rituximab | 500mg | 201.48 | Day 1: 375 mg/m^2^ | 402.96 |
| *Total costs for 1 cycle* | | | | **9985.16** |

| ***“Immune Only”*** | | | | |
| --- | --- | --- | --- | --- |
| ***Tafa-Len*** *(tafasitamab, lenalidomide)* ***– after March 2022***  *- 1 cycle á 28 days, intended for 12 cycles followed by lenalidomide maintenance therapy* | | | | |
| Tafasitamab | 200mg | 793.52 | Cycle 1:  12mg/kg at days  1, 4, 8, 15 & 22 | 19838.00 |
|  |  |  | Cycle 2:  12mg/kg at days  1, 8, 15 & 22 | 15870.40 |
|  |  |  | Cycle 4 onwards:  12mg/kg at days 1 & 15 | 7935.20 |
| lenalidomide | 25mg pills, 1 package á 21 pieces | 611.60 | Day 1-21: 25mg | 611.60 |
| *Total costs for cycle 1* | | | | **20449.60** |
| *Total costs for cycle 2, 3* | | | | **16482.00** |
| *Total costs for cycle 4 onwards* | | | | **85468.80** |
| ***Cellular Therapies*** | | | | |
| ***CAR-T Cells*** | | | | |
| ***Lisocabtagene Maraleucel*** | | | | |
| fludarabine | 250mg | 1638.00 | Day 1-3: 25mg/m^2^ | 918.92 |
| cyclophosphamide | 1000mg | 25.88 | Day 1-3: 250mg/m^2^ | 36.30 |
| lisocabtagene maraleucel |  |  | Day 0:  50 - 110×10^6^ viable  CAR expressing T cells | 345000.00 |
| *Total costs for 1 cycle* | | | | **345955.22** |
| ***Tisagenlecleucel*** *(rituximab, cyclophosphamide, doxorubicin, vincristine, prednisone)  - 1 cycle á 21 days, 6 cycles intended* | | | | |
| FC vor CARs |  |  |  |  |
| fludarabine | 250mg | 1638.00 | Day 1-3: 25mg/m^2^ | 918.92 |
| cyclophosphamide | 1000mg | 25.88 | Day 1-3: 250mg/m^2^ | 36.30 |
| tisagenlecleucel |  |  | Day 0:  0.2 - 5×10^6^  CAR-positive  viable T cells per kg | 320000.00 |
| *Total costs for 1 cycle* | | | | **320955.22** |
